# Nanofibrous materials affect the reaction of cytotoxicity assays

**DOI:** 10.1038/s41598-022-13002-w

**Published:** 2022-05-31

**Authors:** Rafał Podgórski, Michał Wojasiński, Tomasz Ciach

**Affiliations:** 1 Faculty of Chemical and Process Engineering, Department of Biotechnology and Bioprocess Engineering, Laboratory of Biomedical Engineering, Warsaw University of Technology, Waryńskiego 1, 00-645 Warsaw, Poland; 2 Centre for Advanced Materials and Technologies CEZAMAT, Warsaw University of Technology, Poleczki 19, 02-822 Warsaw, Poland

**Keywords:** Nanoscale materials, Nanotoxicology, Biomaterials - cells

## Abstract

Nanofibrous materials are widely investigated as a replacement for the extracellular matrix, the 3D foundation for cells in all tissues. However, as with every medical material, nanofibers too must pass all safety evaluations like in vitro cytotoxicity assays or in vivo animal tests. Our literature research showed that differences in results of widely used cytotoxicity assays applied to evaluate nanofibrous materials are poorly understood. To better explore this issue, we prepared three nanofibrous materials with similar physical properties made of poly-L-lactic acid, polyurethane, and polycaprolactone. We tested five metabolic cytotoxicity assays (MTT, XTT, CCK-8, alamarBlue, PrestoBlue) and obtained different viability results for the same nanofibrous materials. Further, the study revealed that nanofibrous materials affect the reaction of cytotoxicity assays. Considering the results of both described experiments, it is evident that validating all available cytotoxicity assays for nanofibrous materials and possibly other highly porous materials should be carefully planned and verified using an additional analytical tool, like scanning electron microscopy or, more preferably, confocal microscopy.

## Introduction

One-dimensional, usually 10–1000 nm-thin fibers, called nanofibers, were first electrospun more than a century ago by John Francis Cooley in 1900 or Anton Formhals in 1934^1,2^. The high surface-to-volume ratio and the fact that nanofibers can be obtained from a broad spectrum of polymers and additives^3^ give a chance to prepare a new generation of advanced materials used in filtration techniques and catalytic chemistry, medicine or electronics^4,5^. Nowadays, it is also possible to fabricate nanofibrous materials by other methods, such as self-assembly, solution blow spinning, polymerization or template-based synthesis, and centrifugal spinning^6–8^. Nanofibrous materials can mimic collagen fibers' structural and mechanical properties in living tissues^9^. Synthetic nanofibers can degrade over time and be replaced by natural ECM, and such nanofibers, if properly designed, can stimulate the development of specific cell phenotypes^10^. All of that opens up a whole field of implant research using nanofibrous materials, especially those designed to replace and rebuild damaged tissues, called in situ or in vivo tissue engineering^11^. Nanofibrous materials have found applications as a tool for healing wounds^12,13^, bone and cartilage tissue regeneration^7,14–16^, renewal of heart ^17–19^, or even reconstruction of nerve tissue^20,21^.

How do we know that what we developed is a safe biomaterial? Every biomaterial and medical device must first undergo a nonclinical safety valuation that may include cytotoxicity and biocompatibility testing (in vitro tests on animal and human cell lines, in vivo tests on animals) before entering a human clinical trial^22–24^. The first step of understanding the biocompatibility of a medical material or device is a nonclinical safety valuation, which is a long and complex process; even the word "biocompatibility" must be used with caution and proper understanding of the biomaterial-tissue system ^25^. However, multiple simple, rapid, and economic cytotoxicity tests can help determine if the biomaterial has potentially biologically harmful properties or contains toxic substances ^26^.

On the other hand, some publications treating nanofibrous materials' properties contain inadequately detailed descriptions of cytotoxicity analysis procedures. Lack of details includes using no standardized cell lines^27,28^, or methods of nanofibrous material immobilization in culture dish^14,29,30^. For example, because of highly porous structures with open pores, most nanofibrous scaffolds cannot simply sink into the culture medium due to the polymer's hydrophobic properties. They require special inserts to keep them stable and close to the bottom of wells. It is necessary to ensure homogeneous and repeatable cell growth conditions on the surface of the material or its homogeneous extraction for indirect cytotoxicity assays. Further, reports provide different solutions for nanofibrous materials immobilization like steel rings^27,31^, polymer rings^32^ and inserts^33,34^, or just testing without any immobilization^35^—immobilization of material can be another factor influencing materials' cytotoxicity tests results^26^. Another not quite well-explained subject in papers describing cytotoxicity investigation of nanofibrous materials is why one research team decided to use only MTT assay to evaluate cytotoxicity, and another chose XTT assay. (MTT and XTT assays are the most frequently reported methods for cytotoxicity evaluation.) Choosing a cytotoxicity assay is an important issue and should always be adequately described and discussed in papers. The most commonly used metabolic cytotoxicity viability assay—MTT—may sometimes give false positive or false negative results when used to study nanofibrous materials^31^. It happens because, in the MTT assay, mitochondria's reductases produce formazan crystals that are not soluble in water-based media. Then, formazan crystals can be deeply absorbed in the porous structure of nanofibrous scaffolds. After isopropanol or dimethyl sulfoxide (DMSO) extraction, a portion of formazan crystals can stay adsorbed to the nanofibrous materials' surface, which can cause a false positive cytotoxicity outcome^31^. On the other hand, nanofibrous materials' high surface-to-volume ratio can reveal nanofibers' hidden catalytic properties. Some nanomaterials, such as TiO_2_ nanoparticles^36^ or carbon nanotubes^37,38^, can also reduce tetrazolium salt to formazan by itself, without cells present, leading to false-negative results for cytotoxicity assay. Significant interactions with cytotoxicity assays were also observed among other highly porous materials proposed for medical usage. Barnes et al. report that highly porous medical carbon absorbents, used in extracorporeal therapies, can absorb proteins and other media components, resulting in false-positive cytotoxicity results even with extracts cytotoxicity assays^39^. Another example of such material is porous silicon microparticles, proposed as a drug carrier. Laaksonen et al. described a spontaneous reduction of MTT to formazan on the surface of silicon microparticles^40^. The effect of the highly porous surface of biomaterials on cytotoxicity assays may be due to specific chemical groups' presence on the surface. Neufeld et al. investigated the effect of 19 small molecules at six concentrations on cytotoxicity assays like MTT and resazurin. Even a 1 mM concentration of the small molecule in the sample can produce results with deviations of up to 3,000%, which are significantly greater for MTTs than for resazurin-based assays. Neufeld et al. also emphasized that the carboxylic acid and thiol chemical groups are probably the most responsible for reactions leading to cytotoxicity tests' responses without any cell presence ^41^. Hence, although there is a strong recommendation for using at least two different cytotoxicity tests to analyze a new class of materials^37^, we noticed it might not be sufficient.

For this work, we prepared three types of nanofibrous materials made from polymers previously tested by our team and other researchers in the fields of tissue engineering and medical device fabrication: poly-L-lactic acid (PLLA)^42,43^, polyurethane (PU)^44,45^, polycaprolactone (PCL)^46–48^. We purposedly limited our study to synthetic polyesters and polyurethane for clarity and replicability of the presented results. All materials had been obtained by solution blow spinning (SBS) technique^19,49^ (an alternative for electrospinning), and all of them had a similar mean fiber diameter and high porosity. Next, we tested the cytotoxicity of obtained nanofibers by using five readily available cytotoxicity assays based on cells metabolic activity. Three are based on tetrazolium derivatives: MTT XTT, and CCK-8. The other two rely on resazurin: alamarBlue and PrestoBlue. We also tested materials extracts cytotoxicity to ensure that our materials do not release any organic solvents that may remain in the nanofibrous materials after the solution blow spinning process. As an alternative to cytotoxic assays based on cell metabolic activity, we used a Live/Dead staining assay with observation under confocal laser scanning microscopy (CLSM) and scanning electron microscopy (SEM) to view the morphology of cells on nanofibrous scaffolds. Considering the above-presented doubts about cytotoxicity assays for nanofibrous materials evaluation, we decided to check the following hypotheses' correctness: Results of different cytotoxicity tests performed on similar nanofibrous scaffolds differ significantly from each other and also depend on the polymer used for nanofibers production. Hence, all widely used cytotoxicity assays performed to evaluate the same nanofibrous scaffolds should give the output data within the common confidence intervals for all tests.

## Materials and methods

### Reagents

Three polymers commonly used in tissue engineering and medical device fabrication were chosen for this study to produce nanofibrous mats: poly-L-lactic acid (PLLA, Biomer L9000, M_w_ = 200,000 Da, Krailing), polyurethane (PU, Elastollan 1185A, M_w_ = 108,500 Da, BASF), and ε-polycaprolactone (PCL, M_n_ = 80,000 Da, Sigma Aldrich). Solvents for polymers include chloroform, acetone, tetrahydrofuran (Chempur) and 2,2,2-trifluoroethanol (TCI).

The mouse fibroblast cell line L929 (ATCC CCL-1) was purchased from American Type Culture Collection. L929 fibroblasts were maintained in Dulbecco's Modified Eagle Medium (DMEM, Thermo Fisher Scientific) supplemented with 10% fetal bovine serum (FBS, Thermo Fisher Scientific) and antibiotics (100 U ∙ mL^-1^ penicillin, 100 µg ∙ mL^-1^ streptomycin, Thermo Fisher Scientific). Trypsin–EDTA 0.25% solution (Thermo Fisher Scientific) and Phosphate buffer saline without magnesium and calcium ions (PBS, Thermo Fisher Scientific) were used for cell passaging.

Viability/Cytotoxicity assays: MTT (thiazolyl blue tetrazolium bromide, Sigma-Aldrich), XTT (The Cell Proliferation Kit II [XTT], Roche), CCK-8 (Cell Counting Kit-8™, Dojindo Molecular Technologies Inc.), alamarBlue (alamarBlue™ Cell Viability Reagent, Invitrogen), PrestoBlue (PrestoBlue™ Cell Viability Reagent, Invitrogen), Live/Dead (LIVE/DEAD Viability/Cytotoxicity Kit, for mammalian cells, Thermo Fisher Scientific).

Other used reagents: Triton X-100 (Sigma Aldrich), isopropanol (Chempur), ethanol 96% (P.P.H. Stanlab).

### Solution blow spinning and characterization of nanofibrous scaffolds

The solution blow spinning process and methods for characterization of the nanofibrous scaffold are reported in Supplementary Information S1.

### Scanning electron microscopy

Nanofibers sizes, morphology, pore sizes, and morphology of cells cultured on the scaffolds' surface were measured and investigated based on the scanning electron microscopic (SEM, Phenom G1®, PhenomWorld, Netherlands) microphotographs. A rectangular sample was cut and put on the SEM stub using conductive carbon tape from each nanofibrous mat. As prepared, samples were coated with a 15 nm layer of gold (K550X Emitech, Quorum Technologies, UK) to avoid charging of fibers' surface. At least ten randomly chosen spots of each sample were analyzed.

### L929 Cell line culture maintaining

L929 cells were cultured in a DMEM medium with 10% (v/v) FBS, 100 U ∙ mL^-1^ penicillin, 100 µg mL^-1^ streptomycin in 75 cm^2^ cell culture flasks kept at 37 °C in an incubator with 5% CO_2_. The culture was monitored under the microscope every two days, dissociated, and divided when the cell was near 100% confluent. Cell dissociation protocol was based on a trypsin–EDTA solution procedure. Cells concentration was counted in the Thoma cell counting chamber (Marienfeld, Germany).

### Nanofibrous scaffolds extract cytotoxicity

A nanofibrous scaffold made of PCL (n = 3), PLLA (n = 3), and PU (n = 3) was immobilized by polypropylene insert (Fig. S1) in a 24-well plate and sunk in 1.3 mL of supplemented DMEM for 24 h for obtaining scaffolds extracts. Additionally, a sterile solution of 0.1% Triton X-100 in supplemented DMEM was prepared and incubated in the same periods to obtain positive control (n = 3). Supplemented DMEM stored in an incubator for 24 h (n = 3) was treated as a negative control. L929 cell line was maintained in 96-well plates for 24 h in concentration 10^5^ cells ∙ mL^-1^ and 100 μL of culture medium per well. After this time, the DMEM was replaced by extracts. After 24 h of cultivation with extracts, cells were rinsed two times by adding 100 μL of PBS, and following cytotoxicity assays were performed. Detailed protocols for indirect MTT, XTT, CCK-8, alamarBlue, and PrestoBlue cytotoxicity assays are reported in Supplementary Information S1.

In all cases, the relative cell viability was defined as the absorbance/fluorescence ratio from the sample to the absorbance/fluorescence measured for negative control and represented as a mean value ± standard deviation.

### Direct contact cytotoxicity

Nanofibrous scaffolds made of PCL (n = 4), PLLA (n = 4), PU (n = 4) were immobilized by polypropylene inserts (Fig. S1) in 24-well plates. Cells in wells with polypropylene inserts (n = 4) were treated as a negative cytotoxicity control. 1 mL of cell suspension (1 ∙ 10^5^ cells ∙ mL^-1^) was added to each well, and plates were kept for 24 h at 37 °C in an incubator with 5% CO_2_. Before each cytotoxicity assay, the DMEM medium was removed, and each well with cells was rinsed twice with PBS. Detailed protocols for direct MTT, XTT, CCK-8, alamarBlue, and PrestoBlue cytotoxicity assays are reported in Supplementary Information S1.

In all cases, the cell viability was defined as the ratio of the absorbance/fluorescence/ of samples to the absorbance/fluorescence/ of negative cytotoxicity control and represented as a mean value ± standard deviation.

### Proliferation study

A sterile nanofibrous scaffold made of PCL (n = 3), PLLA (n = 3), PU (n = 3) was immobilized by polypropylene insert (Fig. S1) in 24-well plate. To each well, 1 mL of cell suspension (1 ∙ 10^4^ cells ∙ mL^-1^) in supplemented DMEM was added and kept for 24 h, 72 h, and 7 days at 37 °C in an incubator with 5% CO_2_ with exchanging culture medium every 48 h. Cell seeded to wells without scaffolds was treated as negative cytotoxicity control. After each incubation period, the culture medium was removed from the wells, and samples were washed twice with PBS solution to the culture medium.

For the Live/Dead cell viability assay, 2 µM of calcein AM and 4 µM of Ethidium Homodimer-1 part (both parts of Live/Dead assay kit) in PBS solution were added in a volume of 200 µL directly to each well and incubated 30 min in 37 °C. After staining, wet scaffolds were put between two microscopic slides, and the whole surface of each scaffold was scanned by confocal laser scanning microscopy (CLSM, LSM 880, Zeiss, Germany). To account for the uneven distribution of cells, central areas of 4.3 × 4.3 mm for each sample were observed for statistics. A full-area image was taken for each material as proof of even cell distribution. Control was scanned by putting a 24-well plate into CLSM. For each sample, the obtained image was converted in ImageJ software^50^ into two binary images—one for each used dye, then watershed for cell separation, and finally, cells were counted by particle analyzer.

For SEM observation, samples were sunk in freeze cold (− 15 °C) methanol for 15 min to preserve cells on the scaffolds' surface. Then, to remove water, methanol-soaked scaffolds were sunk (5 min each step) in a cascade of EtOH solutions in distilled water with a concentration from 50% (first step) and a growing concentration of EtOH every 10% to the most concentrated 98% solution of EtOH. The dehydrated scaffolds were air-dried and prepared for SEM observation as described in the previous section of the paper.

MTT, XTT, Cell Counting Kit-8, alamarBlue, and PrestoBlue viability assay procedures were the same as described in the direct contact cytotoxicity section above.

### Live/Dead direct contact cytotoxicity

Nanofibrous scaffolds made of PCL (n = 4), PLLA (n = 4), PU (n = 4) were immobilized by polypropylene inserts (Fig. S1) in 24-well plates. Cells in wells with polypropylene inserts (n = 4) were treated as a negative cytotoxicity control. 1 mL of cell suspension (1 ∙ 10^5^ cells ∙ mL^-1^) was added to each well, and plates were kept for 24 h at 37 °C in an incubator with 5% CO_2_. Before cytotoxicity evaluation using Live/Dead, DMEM medium was removed, and each well with cells was double rinsed with PBS. Next Live/Dead cell viability assay, 2 µM of calcein AM and 4 µM of Ethidium Homodimer-1 part (both parts of Live/Dead assay kit) in PBS solution were added in a volume of 200 µL directly to each well and incubated for 30 min in 37 °C. After staining, wet scaffolds were put between two microscopic slides, and the whole surface of each scaffold was scanned by CLSM. Negative cytotoxicity control was scanned by putting a 24-well plate into CLSM. For each sample, the obtained image was converted in ImageJ software into two binary images – one for each used dye, then watershed for cell separation, and finally, cells were counted by particle analyzer. In all cases, the cell viability was defined as the ratio of the counted live cells of samples to the live cells of negative cytotoxicity control and represented as a mean value ± standard deviation. The contrast and brightness of the obtained images have been improved for better visibility of dead cells.

## Results

### Nanofibrous scaffolds properties

**Figure 1:**
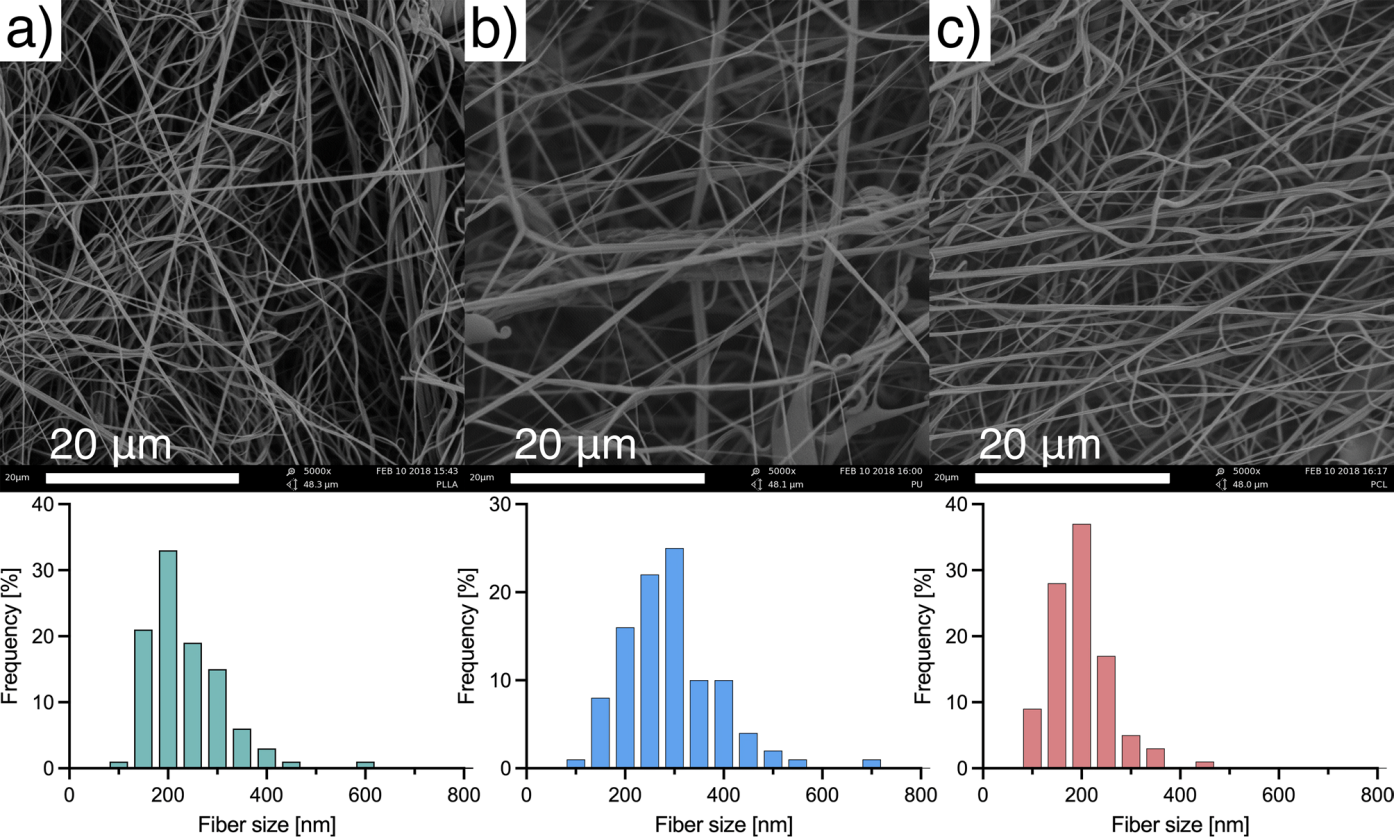
SEM images and fibers size distributions of (**a**) – PLLA; (**b**) – PU; (**c**) – PCL.

Images taken with SEM confirmed the nanofibrous structure of the obtained scaffolds (Fig. 1). The mean fiber diameter, mean pore size, porosity, and water contact angle (Fig. S2) of the material for each polymer are presented in Table 1. The fiber size analysis shows that the size distribution of nanofibers within each of the materials taken for the study remains similar, even though the mean fiber diameter of polyurethane fibers is significantly higher (*p* < 0.001) than the mean sizes of the other nanofibrous materials. Regardless of the differences in polymer–solvent systems we used in our study, produced materials exhibit similar structural properties.

**Table 1:** Mean fibers diameter, nanofibrous scaffolds pore size, and porosity.

Polymer	Fibers mean diameter ± SD [nm]	Mean pore size ± SD [μm]	Porosity ± SD [%]	Water contact angle ± SD [º]**
Poly-L-lactic acid (PLLA)	232 ± 80	2.40 ± 0.82	92.5 ± 1.1	128.8 ± 3.2
Polyurethane (PU)	292 ± 97*	2.74 ± 1.08	83.3 ± 1.2	124.9 ± 1.3
ε-Polycaprolactone (PCL)	199 ± 61	2.44 ± 0.86	77.4 ± 3.1	133.7 ± 0.7

*Sample differs from the rest with p < 0.001.

**Water contact angle values differ from each other with p < 0.001.

### Cytotoxicity

**Figure 2:**
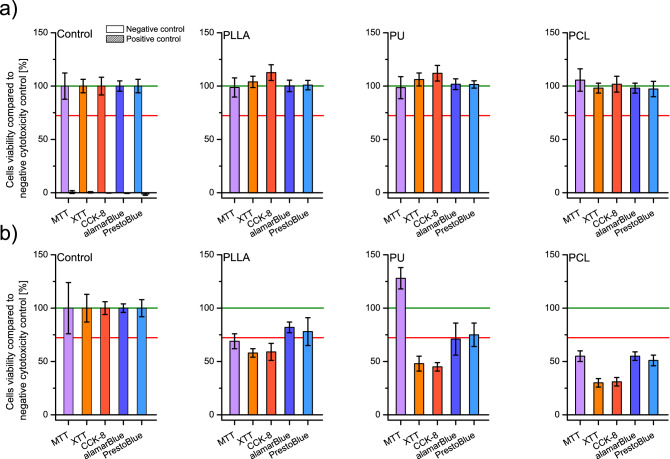
Cells viability was tested by MTT, XTT, CCK-8, alamarBlue, and PrestoBlue assays (**a**) on scaffolds' liquid extracts after 24 h of incubation with PLLA, PU, and PCL; and (**b**) after 24 h of direct culture on PLLA, PU, and PCL scaffolds; including controls. In every graph, the green line represents the negative control result, and a red line represents the threshold for cytotoxicity level of 70% of cells viability compared to the negative control.

All performed cytotoxicity tests carried out on 24 h extracts according to the ISO EN 10993–5 protocol's criteria (a viability value of cells in contact with extract or sample should be < 70% of the negative control to recognize cytotoxic potential) showed no cytotoxicity for every investigated nanofibrous material (Fig. 2a). The obtained results oscillated around 100% of value for control for all materials and all assays, but for the MTT test, the deviations were noticeably higher than for the other tests. Extracting cytotoxicity results proves that no toxic substances are released from the nanofibrous scaffolds, and organic solvent remains used to obtain polymer solutions for spinning must be minimal.

Direct cell culture on materials was chosen to evaluate the influence of scaffolds' physical properties on the L929 cell line. As in the cytotoxicity on extracts method, viability should be reduced to < 70% of the negative control to recognize the cytotoxic potential of tested materials during direct contact between cells and the materials. Direct cytotoxicity assays showed mixed results (Fig. 2b). For PLLA materials, only the alamarBlue and PrestoBlue tests revealed no cytotoxicity giving 82 ± 5% and 78 ± 13% of control, respectively. For PU, the alamarBlue and PrestoBlue tests showed no cytotoxicity giving 71 ± 15% and 75 ± 11%, respectively, and for the MTT test, the result was 128 ± 11% of the control result. All tests showed cytotoxic properties for materials obtained from PCL, and the lowest result was achieved with the alamarBlue test (55 ± 4%). The results for each material show an apparent difference between the test results from different groups: tetrazolium salts which give water-insoluble products (MTT), tetrazolium salts which give water-soluble products (XTT, CCK-8), and resazurin-based tests. The results for alamarBlue are higher than the results of XTT and CCK-8 by 24 ± 1 pp for all materials, and the results for PrestoBlue are higher than the results of XTT and CCK-8 by 23 ± 4 pp. The MTT results for PLLA and PCL are like those of alamarBlue and PrestoBlue; however, 128 ± 11% of control was obtained for PU materials, which would indicate the proliferative properties of the tested PU scaffolds – or interaction between MTT and PU materials itself.

### Cells proliferation

To verify whether the cytotoxicity results after 24 h of direct cell culture are the effect of the cytotoxic properties of the investigated nanofibrous materials themselves or possibly the extended adaptation phase of cells, we decided to carry out the direct culture for 1, 3, and 7 days. Subsequently, the samples were examined after each period using a cytotoxicity procedure with MTT, XTT, CCK-8, alamarBlue, PrestoBlue assay, SEM observation, and Live/Dead staining, followed by observations using CLSM.

#### Metabolic assays

**Figure 3:**
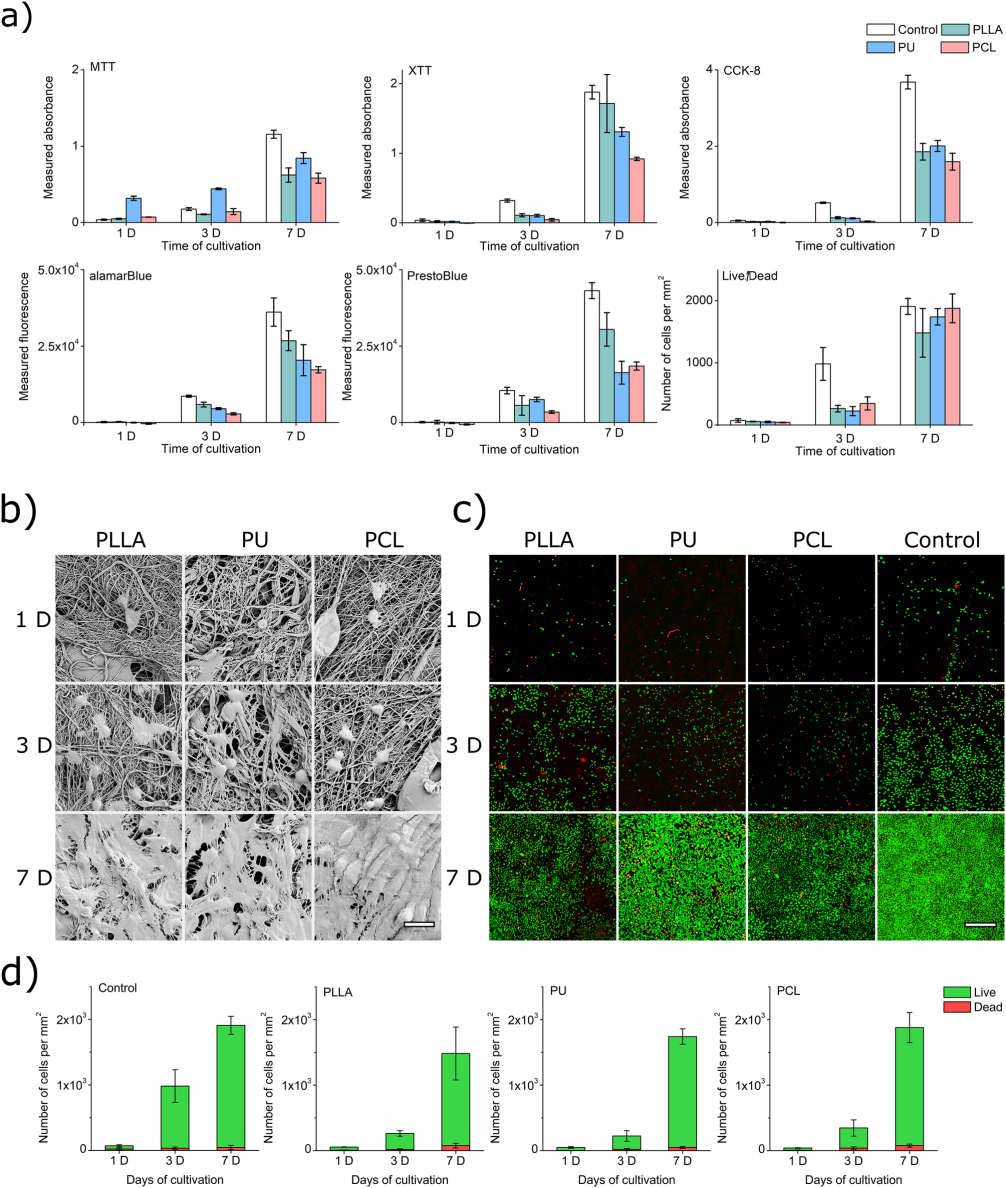
(**a**) Dependency between the result of MTT, XTT, CCK-8, alamarBlue, PrestoBlue, and Live/Dead assay and time of cell cultivation on PLLA, PU, and PCL materials. (**b**) SEM images of L929 cell culture on surfaces of PLLA, PU, and PCL scaffolds after 1 day, 3 days, and 7 days of cultivation. Scale bar represents 20 µm (**c**) CLSM images of L929 cell culture on surfaces of PLLA, PU, and PCL scaffolds after 1, 3, and 7 days of cultivation. Green dots are live cells; red dots are dead cells. The scale bar represents 500 µm. CLSM images of cells on the entire surfaces of the scaffolds are presented in Supplementary Materials (Fig. S4). (**d**) Results of counting live and dead cells in CLSM images of L929 cell culture on surfaces of PLLA, PU, and PCL scaffolds after 1, 3, and 7 days of cultivation.

All assays used for the proliferation study showed a dynamic growth of L929 cells on PLLA, PU, and PCL samples, especially between the 3rd and 7th day of cultivation (Fig. 3a). Observed cell viability was lower than in cytotoxicity control cultures except for PU in MTT assays, probably caused by a previously undescribed reaction between tested materials and MTT tetrazolium salt. However, the results for the 1^st^ day of culture can be problematic due to the sensitivity of the tests. For example, the results were obtained for PU in the case of alamarBlue and PrestoBlue and PCL in the case of XTT, alamarBlue, and PrestoBlue with values lower than for results from empty wells made for obtaining the background. In this situation, removing background from data gave negative results of absorbance/fluorescence visible in the chart. This means that the tests may be too insensitive for such a small number of cells on nanofibrous materials, and perhaps some of the dyes are absorbed on the material's surface.

Recuring high MTT readings prompted us to repeat the experiment with nanofibrous scaffolds and MTT in pure DMEM solution but without cells. Results are presented in Supplementary Materials (Fig. S3). A dense concentration of adsorbed formazan was observed on PU scaffolds after 4 h of incubation in MTT/DMEM solution, and the lowest concentration of formazan was observed on PCL scaffolds (Fig. S3A-C). The high surface-to-volume ratio of nanofibrous scaffolds increased the MTT/scaffolds interactions because, after 24 h of incubation, a similar result appeared on PU granulate after 24 h of incubation (Fig. S3D-F). This behavior can be especially treacherous during proliferation experiments when cytotoxicity assays are used to estimate cell growth. In our case, PU scaffolds generate a much higher absorbance of formazan solution than formazan solutions from other tested materials – which can be erroneously considered more significant proliferation.

#### SEM observations

SEM observation of samples (Fig. 3b) indicated that all materials are suitable for colonization by L929 cells. The difference between images taken after 24 h of culture and 72 h of culture is not significant under visual observation. Still, pairs of cells, probably after division, are visible in samples after 72 h culture. In samples after 7 days of cultivation, we observed nearly 100% coverage of every scaffold's surface with cells. The results show that nanofibrous materials made from PLLA, PU, and PCL are not cytotoxic enough to inhibit or stop the growth of all cells. Moreover, these materials sustain L929 cell growth until cells form a monolayer covering the entire material's surface.

#### CLSM observations

SEM observation does not distinguish which cells are alive and which are dead. We performed Live/Dead assay staining of cells cultured in the same cell culture conditions and periods as for SEM observations to obtain this information. Cell proliferation was observed in all cases. For the 3rd day of culture, the number of cells in the control wells was significantly higher than in tested materials. Viability results for control in metabolic cytotoxicity assays and Live/Dead quantification follow the same pattern (compare Fig. 3a and d) as in SEM images. The L929 cells overgrew each scaffold after 7 days of cultivation (Fig. 3c). On the 7th day of cultivation concentration of cells was about 27 times higher than on the 1st day for control wells. Tested materials showed a similar increase in cell concentration: 27 times higher for PLLA, 37 times higher for PU, and 47 times higher for PCL than the cell concentration on the 1st day for each material. Thus, even with a slower growth rate of cells on the 3^rd^ day of culture on nanofibrous materials, after 7 days, cells cultured on tested materials reached the growth rate of the control culture on tissue culture plastic (TCP).

The scaffold made of PCL nanofibers proved to be the most suitable, among the tested materials, for the survival and growth of L929 cells in all investigated cultivation periods. The percentage of dead cells (Fig. 3d) after the first day of culture was the lowest for PU – 5 ± 1%, with 13 ± 7% for PLLA, 16 ± 9% for PCL, and 31 ± 21% for control. After 3 days of cultivation, only PCL samples had more than 10% (11 ± 7%) share of dead cells. After 7 days of cultivation on all investigated nanomaterials, the highest percentage of dead cells was for PLLA with 5 ± 3%.

### Live/Dead assay for 24 h direct cytotoxicity evaluation

**Figure 4:**
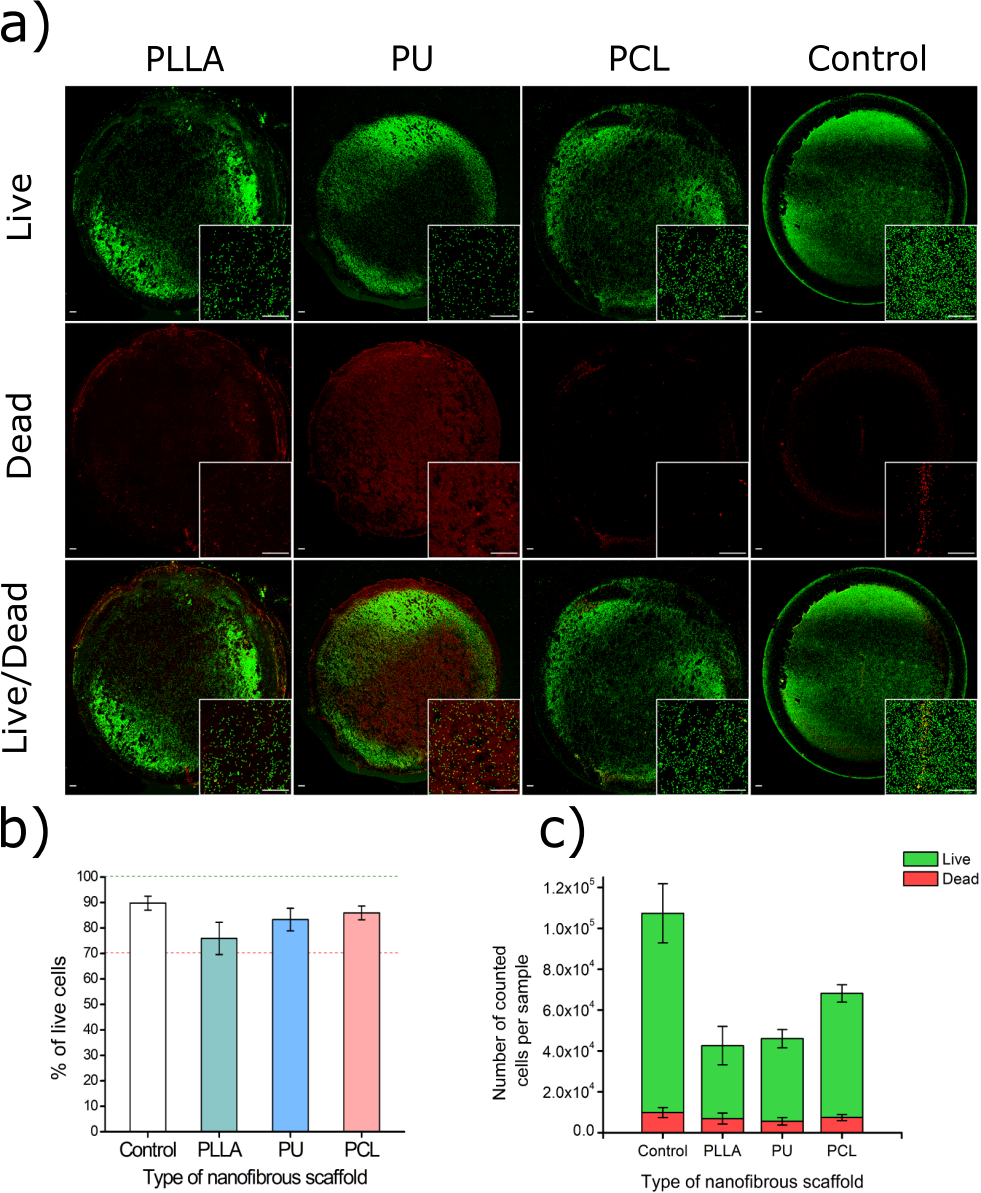
(**a**) CLSM images of L929 cell culture on surfaces of PLLA, PU, and PCL scaffolds after 24 h of cultivation. Green dots are live cells; red dots are dead cells. The scale bar represents 500 µm. (**b**) Population of live cells presented as a percentage of all counted cells. **c)** Population of live and dead cells on each scaffold.

The similarity of the results between nanofibrous materials from the CLSM observations of Live/Dead stained cells inspired us to perform a similar 24 h direct cytotoxicity test with Live/Dead staining. We scanned an area of 17 × 17 mm^2^ for each sample, showing the entire surface of scaffolds (Fig. 4a). For greater readability, 4 × magnification of the central fragment of each scaffold was included. As in the experiments described earlier, the number of cells in the scaffolds is lower than in control (Fig. 4c). In all cases, the population of living cells is above 70% of all cell population: 76 ± 6% for PLLA, 83 ± 4% for PU, 86 ± 3% for PCL, and 90 ± 3% for control (Fig. 4b).

## Discussion

During direct cytotoxicity studies of tested nanofibrous materials, we noticed that when determining cytotoxicity with MTT assay, the results unexpectedly showed high cytotoxicity of PLLA and PCL nanofibrous materials. At the same time, the absorbance result of the MTT test for PU significantly exceeded that of the negative control. We observed significant differences in results in the extended experiment with MTT, XTT, CCK-8, alamarBlue, and PrestoBlue. Such observation shows that there must be additional interaction at the material-assay level.

We first performed cytotoxicity tests on 24 h extracts using five commercially available metabolic cytotoxicity tests to test the hypothesis mentioned above. Extracting cytotoxicity results, oscillating around 100% of control, proves that no toxic substances are released from the nanofibrous scaffolds, and the remnants of the organic solvent used to obtain polymer solutions for spinning must be minimal or nonexistent. This is in line with our expectations and the body of knowledge in the literature—even a direct deposition of fresh-made nanofibers (from SBS) on open wounds is proven safe^51,52^. Similar indirect cytotoxicity results for PLLA, PU, and PCL nanofibrous scaffolds suggest that scaffolds do not release any substance that can interfere with the MTT, XTT, CCK-8, alamarBlue and PrestoBlue cytotoxic assay.

In extract cytotoxicity, the possible interactions between nanofibrous materials and cytotoxicity assays reagents are limited because of the lack of physical contact. Direct culture cytotoxicity tests produced quite different results. For all materials and all tests—except for PU nanofibers and the MTT test—the results were lower than for the control and at the levels indicating the cytotoxicity of the materials. Moreover, different tests have produced mixed results for the same materials. The difference in values between methods used for cytotoxicity evaluation is problematic. It confirmed our hypothesis that nanofibrous scaffolds could lead to different results when subjected to the different in vitro metabolic cytotoxicity assays. Using only one cytotoxicity assay could hide cytotoxicity of scaffolds or viability of cells and, like in the case of PU scaffolds tested with MTT assay, where much higher absorbance of formazan solution was generated than the absorbance of formazan solutions from other tested materials. Moreover, there is a visible division of tests used in the present study into three groups of tests: 1) tetrazolium salts which give water-insoluble products (MTT); 2) tetrazolium salts which give water-soluble products (XTT, CCK-8); and 3) resazurin-based tests (PrestoBlue and alamarBlue). PrestoBlue and alamarBlue cell's viability output is higher than in tests based on tetrazolium salts which give water-soluble products—like XTT or CCK-8. MTT cytotoxicity assay for PLLA and PCL gave results with a value between XTT/CCK-8 and alamarBlue/PrestoBlue, but for PU, results overgrow other tests results by 50 pp. Resazurin, a base compound of both alamarBlue and Presto Blue assays, can be reduced by more mitochondrial enzymes and accept electrons from NADPH, FADH, FMNH, NADH molecules. In contrast, MTT and other assays based on tetrazolium salts work only with mitochondrial oxidoreductase and accept electrons only from NADH molecules^53^. This difference has a high impact on assays sensitivity and can be exposed by the influence of physicochemical properties of tested nanofibrous materials like hindered diffusion or reaction of materials with assay reagents. The difference in MTT, XTT, and CCK-8 cytotoxicity assays can also be caused by the interference of nanofibrous scaffolds with compounds of tests. Similar problems with cytotoxicity assays were observed with other high-porous materials like medical carbon absorbents^39^ or silicon microparticles for drug delivery systems^40^. They may be caused by an increased exposition of functional chemical groups which can react with cytotoxicity assays components ^41^. We postulate that in the case of our nanofibrous PU materials, the high surface-to-volume ratio may enhance the availability of chemical groups of the polymer that can react with cytotoxicity assays – which is similar to the results and conclusion of Neufeld et al.^41^.

The direct cytotoxicity determination results using MTT, XTT, CCK-8, alamarBlue and PrestoBlue assays visibly differ among tested polymeric nanofibrous materials. Although each test response character remains similar for each polymer (except for MTT and PU), the response value varies. The highest viabilities compared to control appear for one of the tested polyesters—PLLA, while the other polyester—PCL exhibits the lowest viabilities. Thus, there is no correlation between the type of the polymer and the direct cytotoxicity results. The different availability of the functional groups in the tested polymers probably affects the adsorption of the output colorants from each cytotoxicity assay^54^. The observed effect adds another limitation to the application of tetrazolium salts and resazurin-based assays for the direct cytotoxicity determination of polymeric nanofibrous materials.

However, insufficient literature data about the observed effects inspired us to analyze the behavior of L929 cells on polymer nanofibrous scaffolds by using direct methods. Even more preferably, we also decided to confirm the lack of cytotoxicity by long-term proliferation (1, 3, and 7 days) and using cytotoxicity assays, SEM observation, and Live/Dead staining assay with confocal microscopy.

In the direct proliferation study with cytotoxicity assays, PU shows the highest responses in the MTT test, regardless of the time point in the study. However, in this case, all results, regardless of the polymer, are lower than those for the TCP. In the proliferation study with XTT, the PLLA nanofibrous materials generate the most variation of the result for the 7th day of culture, showing the highest proliferation. Still, all polymers show lower absorbance than the control. Only the CCK-8 test in the proliferation study gives a uniform response for all tested polymeric nanofibrous materials. However, all absorbance readouts are still about two times lower than those for control on the 7th day of the study. In the resazurin-based test—alamarBlue, the dependence of the test response in the proliferation study reflects the results from the direct cytotoxicity test. This means the highest fluorescence readout appears for PLLA, while the lowest for PCL. Meanwhile, in the PrestoBluse test, the response is mixed, especially on the 7th day of the proliferation study. Again, both resazurin-based tests render the fluorescence lower than measured for TCP. Thus, in the proliferation study, we confirmed a variation in the cytotoxicity studies response depending on the polymer used in the fabrication of nanofibrous—which may create dye-material interaction, especially adsorption interference, as in the case of Jaio et al. work^55^. We identified another limitation of such an approach—the response from the tested materials is always lower than the control on tissue culture plastic (TCP).

Some results obtained for samples analyzed using alamarBlue, and PrestoBlue assays showed negative values because fluorescence values were lower than the background when the cell proliferation was measured. This odd behavior of tests based on resazurin, which should be more sensitive to cell presence than assays based on tetrazolium salts^53^, can be explained by the adhesion of resazurin and resorufin dyes to nanofibrous materials. Similar behavior was described for MTT in Qi et al. work^31^ and might also cause lower results observed in XTT and CCK-8 assays.

Proliferation results obtained in SEM and CLSM showed that cells grow on all materials tested in our study. Moreover, those mouse fibroblasts can cover the surface of each material in 7 days. This is an expected result for non-toxic materials. Similar cell density on nanofibrous materials after 72 h of cultivation was observed by Qi^31^, and full coverage of nanofibrous scaffolds by cell monolayer after 7 days of cultivation was described by Wei et al.^56^, Gao et al.^57^ and Lim et al.^58^. Compared to control, slower growth of L929 cells on scaffolds can significantly impact the results of cytotoxicity tests in cultures in direct contact, contributing to false-positive cytotoxicity results, especially when the test is done in a short contact time, below 3 days. High dead cell percentage after the first day of culture can be explained by higher stress for freshly seeded cells. Despite a higher concentration of dead cells on the 1^st^ day of cultivation, the total share of dead cells in culture decreased for all materials, achieving the lowest results for the 7^th^ day of cultivation. Presented results indicate the possible presence of a prolonged adaptation phase for cells growing on nanofibrous scaffolds—and it is visible in all assays we used: MTT, XTT, CCK-8, alamarBlue, PrestoBlue, and in images of Live/Dead stained cells. Although cell adhesion and growth mechanism depend on nanofibers' material morphology, the diameter of fibers, or even the layout of the scaffold ^59,60^, we observed a significant difference in an adaptation in our study of fibroblast cells to the surface of scaffolds between the control flat surface and tested nanofibrous scaffolds. Nevertheless, for all tested scaffolds after 3 days and 7 days, the share of dead cells remains lower than 15% of all counted cells, which can be found to prove the lack of cytotoxic properties of tested nanofibrous materials. Importantly, results from the Live/Dead direct proliferation study show no visible difference in the test response as a function of the polymer used in nanofibrous materials production.

The obtained results prompted us to consider Live/Dead as a cytotoxicity test method for 24-h incubation of cells with the material. The Live/Dead cell staining method and cell counting using confocal microscopy allow determination of how many cells are on the surface of each tested material and immediately show how many of those cells are alive and how many are dead, which means that one can avoid comparing the absorbance/fluorescence readings of a sample to the negative control, which is the basis of metabolic assays like MTT, XTT, CCK-8, alamarBlue and PrestoBlue. This also solves the problem of the influence of the material on the test components themselves because they are based on a different class of enzymes, in the case of calcein – esterases—and in the case of ethidium-1 homodimer—the staining works to stain the DNA of dead cells. If the material reacted with these reagents, it would be immediately detectable in the images. There is no visible difference among tested polymeric nanofibrous materials in the Live/Dead assay. Hence, the direct determination of cytotoxicity remains unaffected by the material composition, as in the proliferation study mentioned above. Experiment results show that all the nanofiber materials are not cytotoxic, assuming 70% as a threshold for cytotoxicity.

For all quoted research papers about nanofibers, we found only one presented by Balakrishnan et al. ^32^ containing a fully replicating the experiment with another cytotoxicity/proliferation assay. However, we looked more deeply for research papers concerned with producing new nanofibrous materials that contained two or more cytotoxicity assays. We found only a few research papers that meet that criteria from a group of over a thousand works describing cytotoxicity of biomaterials investigation^32–34,61,62^. Such a small number of publications, where the authors verified their experiments with another cytotoxicity assay, may also severely impact the reproducibility observed in natural sciences^63^.

On this basis, we believe that this publication may be the first to broadly describe the issue of differences in results of cell-based assays on polymer nanofibers materials. Many possible factors cause this anomaly in the results, like dye sorption^31^ or nanomaterials' possibility of reducing tetrazolium salt to formazan by^37,64^. To get around this problem, we presented a 24 h direct cytotoxicity assay with Live/Dead staining useful for validating cytotoxicity of problematic medical materials, especially materials with high surface-to-volume ratio, like nanofibers, microporous scaffolds, microspheres, etc. We believe that the data and methods presented in this work will help researchers accurately evaluate the cytotoxicity of new materials, especially nanofibrous materials designed as scaffolds for tissue engineering and regenerative medicine.

## Conclusions

Nanofibers can be useful in medicine, but they must pass cytotoxicity tests. Such tests can produce problems due to possible interactions between nanofibrous materials and reactants in metabolic cytotoxicity assays. This paper investigated the possible different responses of popular metabolic cytotoxicity tests: MTT, XTT, CCK-8, alamarBlue, PrestoBlue, and Live/Dead staining performed on nanofibrous materials from three polymers: PLLA, PU, and PCL. Our results confirm that the interaction between nanofibrous scaffolds and cytotoxicity assays is possible.

The indirect and direct cytotoxicity tests were inconclusive and required verification by using different methods—we conducted SEM and CLSM studies that confirmed that L929 cells could grow on all tested nanofibrous materials. The experiments showed that the results might vary because of factors such as nanofibers' effect on the diffusion of substrates and/or products of cytotoxicity tests or cells' prolonged adaptation to the nanofibrous surfaces.

Based on the presented results, we state that a more accurate analysis of cell behavior during the nanofibrous materials testing should be performed than only one metabolic cytotoxicity test, especially for testing a new class of materials. Also, we recommend 72 h as the minimum cultivation time of cells directly on nanofibrous materials prior to conducting a metabolic cytotoxicity test because of the extended adaptation of cells to the new environment. In the case of any divergent results, we suggest using Live/Dead staining combined with confocal microscopy. Based on our experience, using Live/Dead is the most accurate method for cytotoxicity determination. Scanning the entire surface of the sample stained with Live/Dead using a confocal microscope allowed us to obtain quantitative cytotoxicity results, even after 24 h of cell cultivation (although the 72 h cultivation period remains preferable).

We hope that our findings will allow other research teams to plan better and more accurate in vitro experiments associated with nanofibrous materials. Also, the different effects of cytotoxicity tests depending on nanofibers' physical and chemical properties may indicate the possibility of investigating other curious features of such materials. Such details can allow finding nanofiber materials with properties even more suitable for applications such as biomaterials. Further, we recognize that other highly porous materials designed for medical applications may require a similar cytotoxicity test planning and performance as nanofibrous materials.

## Supplementary Information

Supplementary Information.

## Data Availability

The raw and processed data required to reproduce these findings are available to download from OSF https://osf.io/83jf4/?view_only=22064e9f8e0d4ec9a006561e9068802e.
