# Supplementary material for: Nanofibrous materials affect the reaction of cytotoxicity assays
Source: Sci Rep. 2022 May 31;12:9047. doi: 10.1038/s41598-022-13002-w (PMC9156782; doi:10.1038/s41598-022-13002-w)
Supplement: Supplementary file 1 — Supplementary Information. [file 41598_2022_13002_MOESM1_ESM.pdf]

Supporting information for:

Nanofibrous materials affect the reaction of cytotoxicity assays

Rafał Podgórski[1], Michał Wojasiński[1,*], Tomasz Ciach[1,2]

[1] *Warsaw University of Technology, Faculty of Chemical and Process Engineering, Department of Biotechnology and Bioprocess Engineering, Laboratory of Biomedical Engineering, Waryńskiego 1, 00-645 Warsaw, Poland*

[2] *Centre for Advanced Materials and Technologies CEZAMAT, Poleczki 19, 02-822 Warsaw, Poland*

*Corresponding author e-mail address: michal.wojasinski@pw.edu.pl

1. Methods

1.1. Preparation of polymer solutions and solution blow spinning

Solutions for blow spinning were prepared in a mass concentration of 6 weight %. PLLA was dissolved in a mixture of chloroform and acetone (3:1 volume ratio), PU was dissolved in pure tetrahydrofuran, and PCL was dissolved in 2,2,2-trifluoroethanol. Solutions were left under stirring overnight to ensure proper polymer chain conformation leading to viscoelastic properties suitable for fiber production before SBS process.

All polymer solutions were spun using a SBS system described in detail in our previous work[1]. In brief, a polymer solution was loaded into the 20 mL syringe, and the syringe was put on an injection pump (KDScientific, USA). 10 mL of each solution with a 30 mL $\cdot$ h^{-1} feed rate was used in single nanofibrous mat production. Using an air pump (HV01, Hydrovane, UK), 0.1 MPa working pressure of the air was supplied to the SBS nozzles system. Polymer solution and compressed air were fed into the concentric nozzles system to the inner nozzle and an outer nozzle, respectively. The polymer solution jet was formed in the air stream, and a thread was stretched, leading to the fiber's formation. Fibers were collected on the surface of a rotating cylindrical collector with additional reciprocating movement in the collector's axis to ensure uniform thickness of the collected nanofibrous mat. For further experiments, nanofibrous mats were cut into disc-shaped scaffolds with 16 mm in diameter.

1.2. Fibers sizes and distribution

Nanofibers size was measured based on SEM microphotographs using Fiji open-source image processing software (ver. 2.0.0-rc-43/1.51s)[2]. For materials produced from each polymer, 100 fibers were measured, and the mean fiber diameter ± standard deviation was calculated and listed in Table 1. To determine the statistical difference between each nanofibrous material's mean fiber size, the one-way ANOVA test followed by the post-hoc Tukey's test (p=0.01) was performed.

SEM microphotographs were used as a basis for pore size measurement by Fiji software. For materials produced from each polymer, 100 pores area was measured and compared to a circle with the

same area to calculate pore size. Next, the mean pore size ± standard deviation were calculated and listed in Table 1. To determine the statistical difference between each nanofibrous material pore size, the one-way ANOVA test followed by the post-hoc Tukey's test (p=0.01) was performed.

Porosity was measured using gravimetric analysis, reported previously[3]. Briefly, three 16 mm in diameter scaffolds of each polymer nanofibrous materials were analyzed. First, samples were weighed using a high precision scale. Second, the thickness of each sample was analyzed using SEM. The porosity of each material was calculated using the following equation:

$$P[\%] = \left(1 - \frac{m_s/(A_s \times \delta)}{d_p}\right) \times 100\%$$

where:

m_s – scaffold mass [mg]; A_s – scaffold area [mm^2]; δ – scaffold thickness [mm]; d_p – polymer bulk density [g · cm^{-3}]; d_{PLLA} = 1.25 g · cm^{-3}, d_{PU} = 1.12 g · cm^{-3}, d_{PCL} = 1.145 g · cm^{-3}.

Mean nanofibrous scaffolds porosity ± standard deviation was calculated based on described measurements. The results are listed in Table 1 in manuscript.

1.3. Water contact angle measurement

The water contact angle of PLLA, PU, and PCL nanofibrous materials was measured using the Drop Shape Analysis system DSA100 equipped with software ADVANCE (KRÜSS, Germany). Sessile drop measurement was performed using a 5 µL drop of distilled water placed on the nanofibrous materials' surface and observed over 10 seconds, recording water contact angle values each second. The overall water contact angle was calculated as a mean value of the right and left contact angle. Measurements were conducted 5 times for each investigated material (n = 5), and results were reported as the mean value of water contact angle ± standard deviation. To determine the statistical difference between each nanofibrous material water contact angle, the one-way ANOVA test followed by post-hoc Dunn's test (p=0.01) was performed (water contact angle value does not follow a normal distribution, according to Shapiro-Wilk normality test with p=0.05).

1.4. Nanofibrous scaffolds extract cytotoxicity – detailed procedure

For MTT viability assay, a total of 50 µL of MTT in pure DMEM (1 mg · mL^{-1}) solution was added to each culture well and incubated for 4 h. When MTT was reduced to formazan by live cells, the culture medium was removed, and 100 µL of isopropanol was added to each well and stirred. After 15 minutes of dissolving, the absorbance was measured at 570 nm in a plate spectrophotometer (Epoch, BioTek, USA).

For XTT viability assay, 100 µL of DMEM, without phenol red and supplementation, and 50 µL of XTT with electron-coupling reagent solution was added to each culture well and incubated for 4 h. When XTT was reduced to formazan pigment by live cells, the 100 µL of assay medium from each well was transferred to a new 96-well plate, and the absorbance was measured at 475 nm in a plate spectrophotometer.

For CCK-8 viability assay, a total amount of 10 µL of CCK-8 reagent solution and 100 µL of DMEM without phenol red was added to each culture well and incubated for 4 h. When CCK-8 was reduced to formazan pigment by living cells, 100 µL of the assay medium was transferred to a new 96-well plate, and the absorbance at 450 nm was measured in a plate spectrophotometer.

For alamarBlue viability assay, a total amount of 100 µL of alamarBlue solution in pure DMEM (1:9 v/v) was added to each culture well and incubated for 4 h. After the alamarBlue's resazurin was reduced to resorufin dye by live cells, the assay medium was transferred to a black 96-well plate. Plates were checked in a fluorescence reader (Spark 10M, Tecan, Switzerland) with excitation wavelength at 555 nm and emission wavelength at 590 nm.

For PrestoBlue viability, a total amount of 100 µL of PrestoBlue solution in pure DMEM (1:9 v/v), without phenol red and supplementation, was added to each culture well and incubated for 4 h. After the PrestoBlue's resazurin was reduced to resorufin dye by living cells, the assay medium was transferred to a black 96-well. Plates were checked in a fluorescence plate reader with excitation wavelength at 540 nm and emission wavelength at 610 nm.

1.5. Direct contact cytotoxicity – detailed procedure

For MTT viability assay, a total of 500 µL of MTT in DMEM without phenol red (1 mg · mL^{-1}) solution was added to each culture well and incubated for 4 h. When MTT was reduced to formazan by living cells, the culture medium was removed, and 1 mL of isopropanol was added to each well and stirred. After 15 minutes of dissolving, an 800 µL of obtained formazan solution from each sample was transferred to a new 24-well plate, and the absorbance was measured at 570 nm in a plate spectrophotometer.

For XTT viability assay, 1 mL of DMEM, without phenol red and supplementation, and 500 µL of XTT with electron-coupling reagent solution was added to each culture well and incubated for 4 h. When XTT was reduced to formazan pigment by living cells, the 1 mL of assay medium from each well was transferred to a new 24-well plate, and the absorbance was measured at 475 nm in a plate spectrophotometer.

For CCK-8 viability assay, a total amount of 100 µL of CCK-8 reagent solution and 1 mL of DMEM without phenol red was added to each culture well and incubated for 4 h. When CCK-8 was reduced to formazan pigment by live cells, 800 µL of the assay medium was transferred to a new 24-well plate, and the absorbance at 450 nm was measured in a plate spectrophotometer.

For alamarBlue viability assay total amount of 1 mL of alamarBlue solution in pure DMEM (1:9 v/v) was added to each culture well and incubated for 4 h. After the alamarBlue's resazurin was reduced to resorufin dye by live cells, the assay medium was transferred to a black 96-well plate - 400 µL of each sample was divided into four 100 µL portions for 4 smaller wells. Plates were checked in fluorescence reader with excitation wavelength at 555 nm and emission wavelength at 590 nm.

For PrestoBlue viability total amount of 1 mL of PrestoBlue solution in pure DMEM (1:9 v/v) was added to each culture well and incubated for 4 h. After the PrestoBlue's resazurin was reduced to resorufin dye by live cells, the assay medium was transferred to a black 96-well plate - 400 µL of each sample was divided into four 100 µL portions for 4 smaller wells. Plates were checked in fluorescence reader with excitation wavelength at 540 nm and emission wavelength at 610 nm.

2. Figures

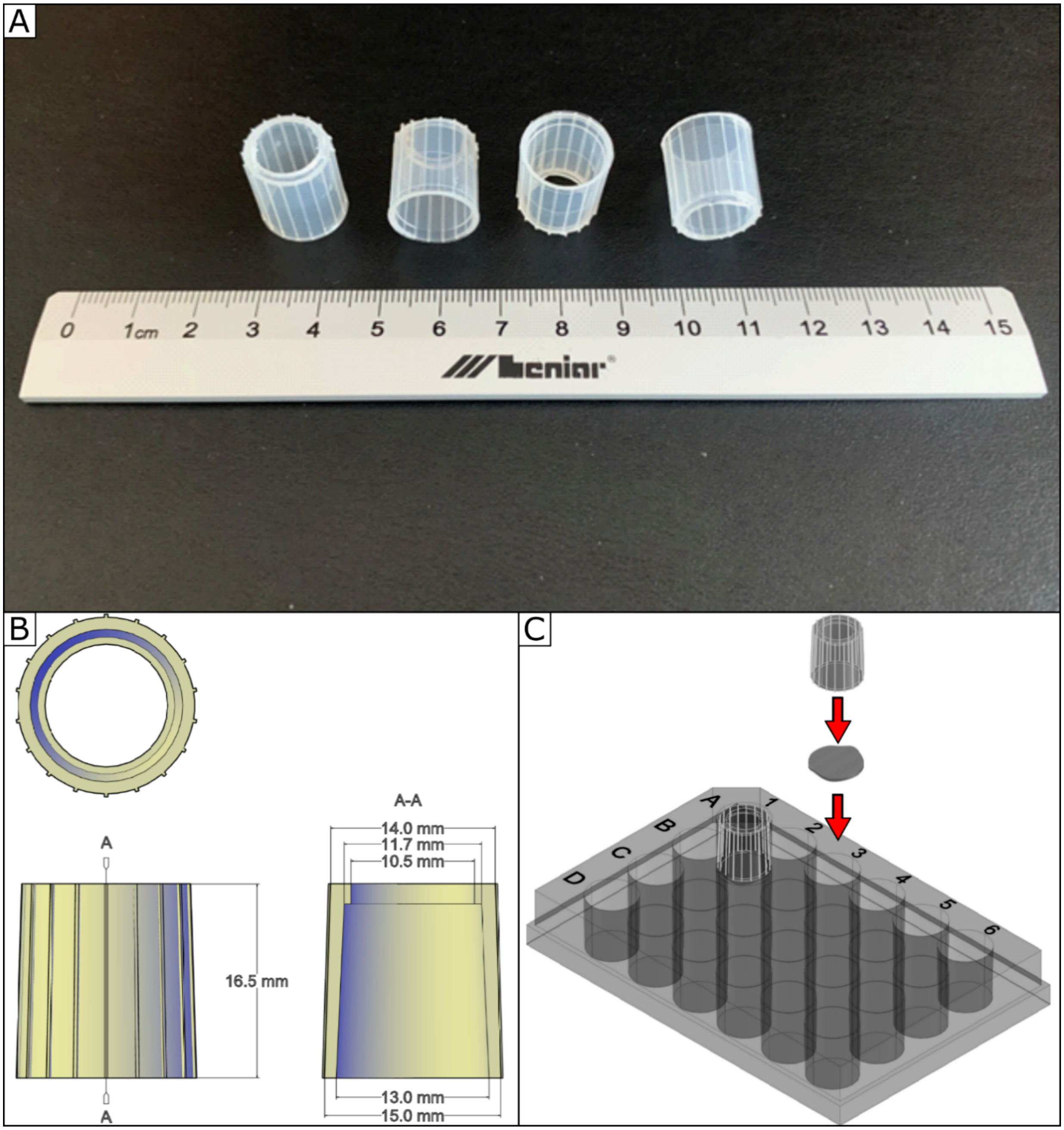

Figure S1. A - Polypropylene inserts used to immobilize nanofibrous scaffolds on a bottom of well in 24-well plate; B – Scheme and dimensions of polypropylene insert; C – Placement of the scaffold in 24-well plate for cytotoxicity and proliferation study.

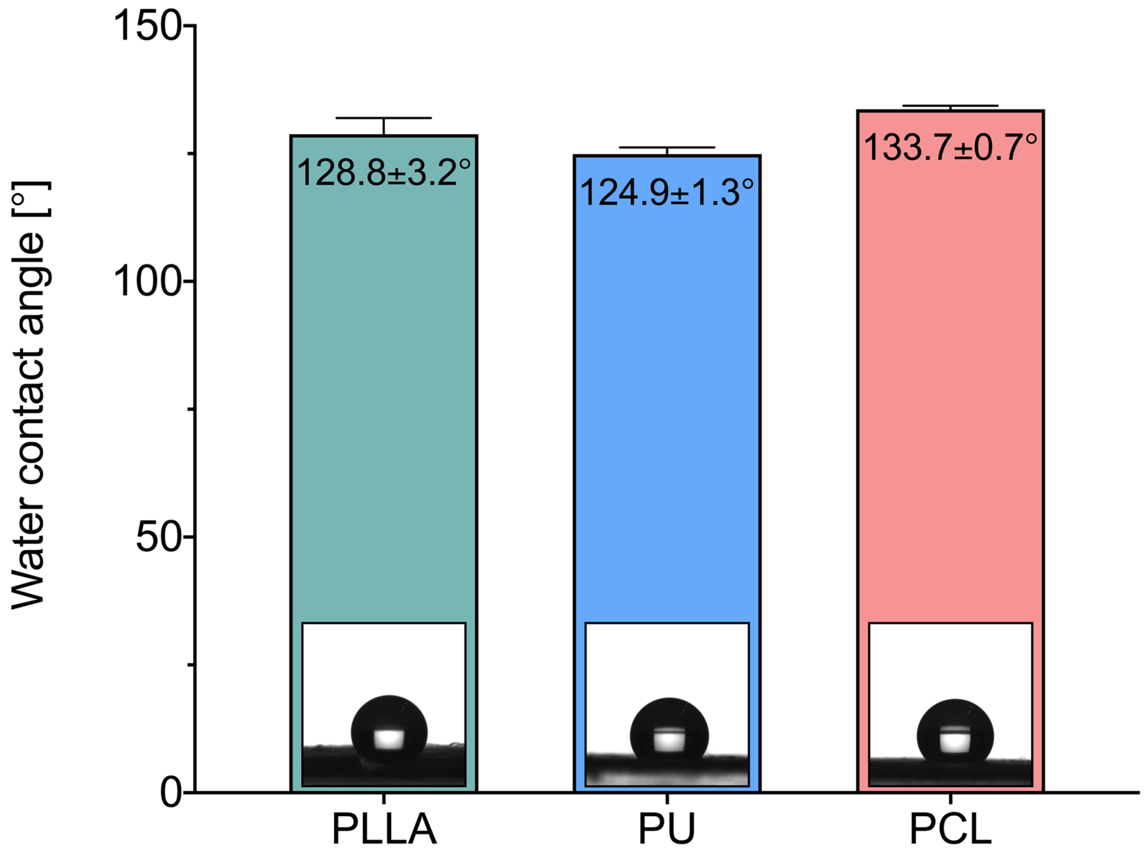

Figure S2. Water contact angle of PLLA, PU, and PCL nanofibrous materials.

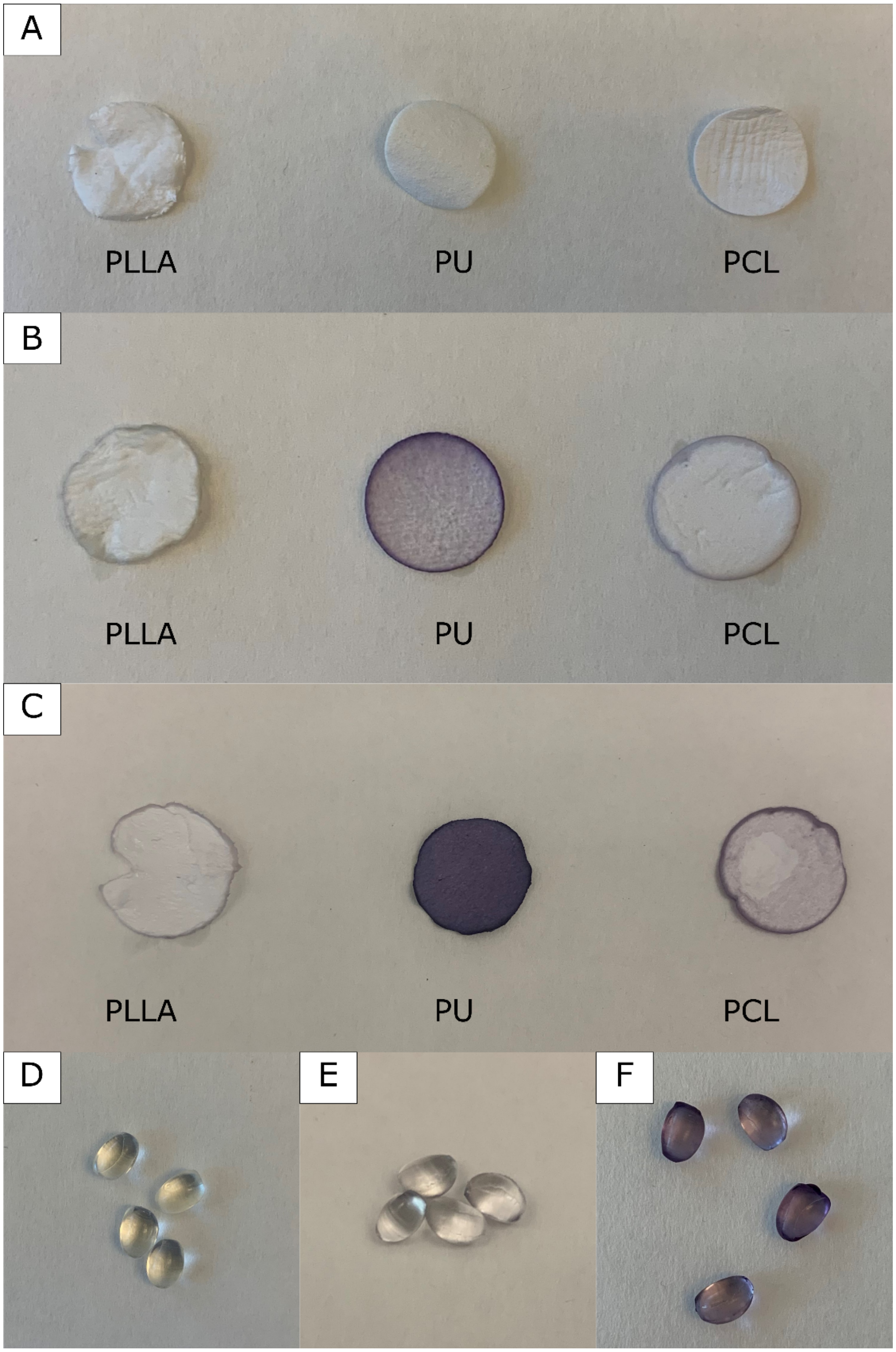

Figure S3. A – PLLA, PU, and PCL nanofibrous scaffolds; B – PLLA, PU, and PCL scaffolds after 15 minutes in MTT solution in DMEM; C – PLLA, PU, and PCL scaffolds after 4 hours in MTT solution in DMEM; D – PU granulate, E – PU granulate after 4 h in MTT solution in DMEM, F – PU granulate after 24 h in MTT solution in DMEM.

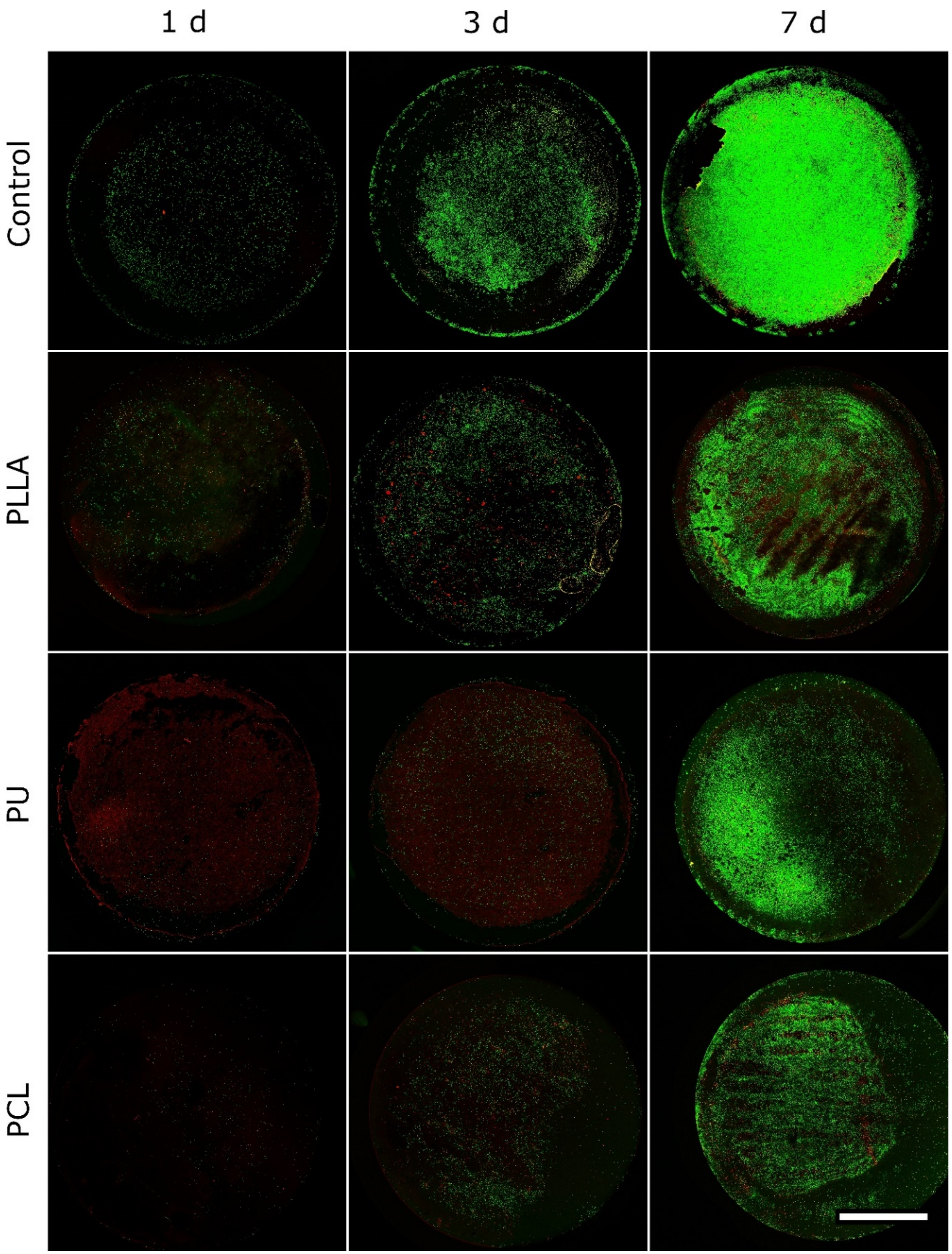

Figure S4. CLSM images of L929 cell culture on surfaces of PLLA, PU, and PCL scaffolds after 1, 3 and 7 days of cultivation. Green dots are live cells; red dots are dead cells. The scale bar represents 5 mm.

3. References

1. Wojasiński, M., Pilarek, M. & Ciach, T. Comparative Studies of Electrospinning and Solution Blow Spinning Processes for the Production of Nanofibrous Poly(L-Lactic Acid) Materials for Biomedical Engineering. *Polish J. Chem. Technol.* **16**, 43–50 (2014).

2. Schindelin, J. *et al.* Fiji: an open-source platform for biological-image analysis. *Nat. Methods* **9**, 676–682 (2012).

3. Tomecka, E. *et al.* Poly(L-lactic acid) and polyurethane nanofibers fabricated by solution blow spinning as potential substrates for cardiac cell culture. *Mater. Sci. Eng. C* **75**, 305–316 (2017).
